# Weight stigma is associated with body mass index among college students in Taiwan: the mediated role of internalized weight stigma

**DOI:** 10.1186/s40359-023-01414-w

**Published:** 2023-11-01

**Authors:** Yi-Ching Lin, Chung-Ying Lin, Mohsen Saffari, Meng-Che Tsai, Yun-Hsuan Chang, Carol Strong, Ji-Kang Chen, Yi-Ping Hsieh, Yung-Ning Yang, Janet D. Latner

**Affiliations:** 1 Department of Early Childhood and Family Education, National Taipei University of Education, Taipei, 106320 Taiwan; 2 Institute of Allied Health Sciences, College of Medicine, National Cheng Kung University, 1 University Rd, Tainan, 701 Taiwan; 3 University of Religions and Denominations, Qom, Iran; 4 Department of Public Health, College of Medicine, National Cheng Kung University, Tainan, Taiwan; 5 Biostatistics Consulting Center, National Cheng Kung University Hospital, College of Medicine, National Cheng Kung University, Tainan, Taiwan; 6 Health Research Center, Life Style Institute, Baqiyatallah University of Medical Sciences, Tehran, Iran; 7 Health Education Department, Faculty of Health, Baqiyatallah University of Medical Sciences, Tehran, Iran; 8 Department of Pediatrics, College of Medicine, National Cheng Kung University Hospital, National Cheng Kung University, Tainan, Taiwan; 9 Department of Medical Humanities and Social Medicine, College of Medicine, National Cheng Kung University, Tainan, Taiwan; 10 Institute of Gerontology, College of Medicine, National Cheng Kung University, Tainan, Taiwan; 11 Department of Psychology, National Cheng Kung University, Tainan, Taiwan; 12 Department of Social Work, Chinese University of Hong Kong, New Territories, Hong Kong China; 13 Department of Social Work, College of Nursing and Professional Disciplines, University of North Dakota, Grand Forks, ND USA; 14 Department of Pediatrics, E-DA Hospital, I-Shou University, Kaohsiung, Taiwan; 15 School of Medicine, I-Shou University, Kaohsiung, Taiwan; 16 Department of Psychology, University of Hawaii at Manoa, Honolulu, HI USA

**Keywords:** Perceived weight stigma, Internalized weight stigma, Changes in BMI, Psychological distress, College students, Obesity

## Abstract

**Background:**

Weight stigma is an issue often studied in Western countries; however, such information is scarce in Asian studies.

**Methods:**

This study aimed to examine the role of internalized weight stigma as a mediator in the relationship between perceived weight stigma and changes in body mass index (BMI). The data were collected through a longitudinal online survey with two phases (n = 974; Phase 1: August and September 2021; Phase 2: November and December 2021). The Perceived Weight Stigma Scale (PWSS), Weight Self-Stigma Questionnaire (WSSQ), and Depression, Anxiety, Stress Scale − 21 (DASS-21) were administered to assess perceived weight stigma, internalized weight stigma, and psychological distress. Hierarchical regressions were used to examine the proposed model, and Hayes’ Process Macro was used to test a mediation model.

**Results:**

The changes in perceived weight stigma and internalized weight stigma were significantly and positively associated with changes in BMI. There were significant and positive associations between perceived weight stigma, internalized weight stigma and psychological distress over time. Change in internalized weight stigma was found to be a significant mediator in the association of change in perceived weight stigma with change in BMI for the entire sample (unstandardized coefficient = 0.04; 95% CI = 0.02, 0.06), female subgroup (unstandardized coefficient = 0.05; 95% CI = 0.02, 0.08), and male subgroup (unstandardized coefficient = 0.03; 95% CI = 0.01, 0.06). Change in perceived weight stigma also had significant effects on change in BMI for the entire sample and the female subgroup, but not for the male subgroup.

**Conclusion:**

Because perceived weight stigma may significantly impact changes in BMI through internalized weight stigma, treatment strategies to ameliorate self-stigma may enhance the results of weight-reduction programs. Such treatment strategies should be considered for inclusion in weight-loss interventions.

## Abstract (plain language summary)

Weight stigma is commonly studied in Western countries rather than in Asian ones. To further explore how internalized weight stigma mediates the relationship between perceived weight stigma and the changes in body mass index (BMI), the study collected data through a longitudinal online survey with two phases by applying the Perceived Weight Stigma Scale (PWSS), Weight Self-Stigma Questionnaire (WSSQ) and Depression, Anxiety, Stress Scale − 21 (DASS-21) to assess perceived weight stigma, internalized weight stigma, and psychological distress. The changes in perceived and internalized weight stigma were significantly and positively associated with changes in BMI. There were significantly positive associations between perceived weight stigma, internalized weight stigma, and psychological distress over time. Based on the findings, treatment strategies to alleviate self-stigma may enhance the results of weight-reduction programs and should be considered for inclusion in weight-loss interventions.

## Introduction

Obesity has doubled across 70 countries over the last 40 years [1], and physical inactivity is one of the most common behavioral factors [2]. Among different age groups, college students are a high-risk group for obesity and physical inactivity, which are, in turn, susceptible to developing non-communicable diseases (NCDs) later in life [3]. A high proportion of college students are characterized by sedentary behavior [4]. They tend to spend most of their time on activities such as studying, sitting in lectures, using computers or cell phones, surfing the Internet, or playing video games, which may expose them to physical inactivity and weight gain. In addition, entering a new challenging chapter of life immediately after adolescence, college students undergo physiological and psychological stressors influencing many aspects of their health behaviors and lifestyles, including nutritional/exercise habits, which could lead to adverse health outcomes such as overweight and obesity [5].

According to a secular trend study investigated across two waves, the Nutrition and Health Survey in Taiwan (NAHSIT 1993–1996 and NAHSIT 2005–2008), the prevalence of overweight and obesity increased in both men and women [6]. Subsequently, another study [7] combined three sets of data from NAHSIT 1993–1996, 2005–2008, and 2013–2016 to examine the prevalence of overweight and obesity. The results indicated an increase in the prevalence of obesity among men but not in women, which was especially significant among young and middle-aged populations. However, the long-term relationship between the escalating prevalence of obesity and its potential risk factors, such as weight stigma, has not been examined in Taiwan.

Many adverse health outcomes due to being overweight or obese may result from personal experience, perception, anticipation, and internalization of weight stigma [8]. Weight stigma delineates negative beliefs, attitudes, denigration, or social castigation directed toward individuals because of their weight [9]. Weight stigma usually manifests through stereotypes, prejudicial attitudes, and discriminatory actions [8, 9]. Two weight stigma constructs are distinctly defined: perceived and internalized weight stigma. Perceived and experienced weight stigma are often applied interchangeably to describe how others think about the individual’s weight. On the other hand, internalized weight stigma refers to accepting societal weight bias as true of oneself [10]. Just as the obesity rate has been increasing in recent decades, weight stigma has also escalated [11] and is increasingly recognized as a global health issue [12].

Perceived weight stigma indicates perceived prejudicial attitudes toward and social devaluation of individuals identified as having excess body weight or not complying with the prevailing thin body ideal [13, 14]. These unjust attitudes are rooted in negative stereotypes, such as judging obese people as clumsy, incompetent, or lacking in determination [15]. For mistreated individuals, perceived weight stigma may elicit physiological and behavioral consequences, intensify declining metabolic health, and thus augment weight gain [9, 16]. High perceived weight stigma is significantly related to impairments and disturbances in mental health [17].

Furthermore, stigmatizing portrayals of obese individuals are pervasively present in the mass media. Characters with larger body sizes are usually depicted with prejudice and devaluation and are seen as associated with stereotypical behaviors such as sedentary behavior or overeating [18]. These images could reinforce arbitrary perceptions that obesity stems from personal choices, which may result in a lower level of public interest in obesity-related preventions or interventions as well as more significant weight stigma among the general public [19].

Perceived stigma may be converted to internalized weight stigma when perpetrators take stigmatizing actions toward their victims [20]. Subsequently, internalized weight stigma would lead to negative health outcomes and mediate the association between perceived weight stigma and health outcomes [21]. Internalized weight stigma is defined as acceptance of negative stereotypes about weight, applying negative stereotypes personally, and devaluing oneself due to self-defining as overweight [22]. Also, internalized weight stigma could be transmitted negatively, such as through societal discrimination or devaluation, and subsequently causes psychosocial problems, including aggressive behavior, lower self-esteem, depression, suicide ideation, and anxiety [23]; on the other hand, internalized weight stigma could be conveyed in seemingly positive ways. For instance, medical advice for weight loss strategies emphasizes that individuals should be responsible for weight management. Thus, medical advice encourages individuals to exert their willpower to exercise more or eat less.

Such encouragements that may be perceived as motivations for weight loss may provoke feelings of shame [24, 25] and self-blame [26], intensifying internalized weight stigma. In addition, given that body image is greatly influenced by social desirability and experiences [27], individuals with larger body shapes tend to have higher levels of body dissatisfaction [28] and body image disturbance [29], which may heighten internalized weight stigma as well. Internalized weight stigma increases vulnerability to psychological distress, and the harm could intensify internalized weight stigma in turn. The long-term consequences of internalized weight stigma include psychological problems and the risk of future weight gain and obesity [30].

Hence, internalized weight stigma may be an unintended consequence of anti-obesity efforts and hinder the intended effect [14]. Also, literature has indicated a gradient relationship between internalized weight bias and body weight [13]. A large body of literature has articulated that perceived weight stigma is related to high weight bias internalization [31] and that perceived and internalized weight stigma influences health outcomes [8, 10, 32]. Different cross-sectional studies have investigated the impacts of weight stigma in general, perceived weight stigma, and internalized weight stigma on various health outcomes [33–36].

Given the link between perceived and internalized weight stigma and health problems mentioned above, further exploration of how weight stigma gradually penetrates individuals’ perceptions and impacts different health outcomes is warranted. However, longitudinal research still needs to prospectively examine the effect of perceived and internalized weight stigma on health outcomes. Furthermore, more is required to know about the progress of individuals perceiving weight stigma, internalizing it, and the potentially sequent impact on health outcomes in a trajectory model. In addition, the scope of this research should be widened and explored in contexts other than just Western culture.

Several research studies have explored the role of internalized weight stigma as a mediator of the association between perceived weight stigma and biopsychosocial outcomes such as psychological well-being and physical health [37], body shame [38], and exercise, but not changes in body mass index BMI, an objective biomarker that plays a decisive role in health [39]. Considering the growing prevalence of obesity worldwide, it is critical to further assess weight stigma. Weight stigma has been found to be associated with increased BMI, such that people with obesity often perceive and internalize discrimination because of their weight. In other words, weight stigma is the consequence of increased BMI [40]. Compared to prior studies in which BMI was controlled to avoid its biological correlate of weight stigma [10, 29, 41], the present study defined body mass index (BMI) changes as a critical health outcome.

In a longitudinal research design, data on changes in BMI were examined to explore the proposed model. Also, most previous weight stigma-related literature was conducted in Western contexts [13, 42, 43]. Thus, exploring the model within Eastern societies is essential. An important strategy in fighting the obesity epidemic involves identifying and addressing predisposing factors.

### Aims

This study aimed to underscore the negative consequences of weight stigma on weight-related health outcomes. In a longitudinal study, this investigation aimed to examine the role of internalized weight stigma as a mediator of the relationship between perceived weight stigma and changes in BMI, controlling for psychological distress.

## Methods

### Participants, data collection, and ethical considerations

The inclusion criteria of the eligibility were (i) studying in a college program; (ii) having the ability to read written Chinese; and (iii) being aged 20 years or above (i.e., a legal age to provide informed consent without a guardian in Taiwan). All participants provided an e-form informed consent to confirm their willingness to participate in the present study. No specific exclusion criteria were used for the eligibility. Moreover, the Institutional Review Board in the Chi Mei Medical Center (IRB Serial No.: 11007-006) and the Human Research Ethics Committee in the National Cheng Kung University (Approval No.: 109-551-2) approved the study.

The data collection was conducted using *Google Forms*. More specifically, a link and a QR code connected to *Google Forms* were distributed to the target participants with the help of the authors’ colleagues teaching or studying at a university in Taiwan. The link was then shared among the participants interested in the present study; the participants were informed that they could forward the link to their university student friends. Due to the nature of the snowballing sampling method, the current authors could not trace all the distributed links and were unable to calculate the response rate. Therefore, the authors only set a required sample size for the present study at baseline (i.e., 1000 participants).

After logging in to *Google Forms*, each participant could read the study purpose, participant eligibility, incentive information (about 3.3 USD were provided for each time of participation), and participant rights in detail. Then, they were asked to click the ‘agree’ button to represent their e-form informed consent if they wanted to participate. If the participant did not want to provide e-form informed consent, they were advised to click the ‘disagree’ button, and the survey ended. Those who clicked the ‘agree’ button could read the survey questions and responses. Also, the participants could discontinue the online survey at any time. The participants were asked to provide their email addresses and mobile phone numbers at baseline for the authors to follow up with them three months later. The baseline survey was conducted between August and September 2021, and the follow-up was conducted between November and December 2021. Although the data collection periods (including both baseline and follow-up surveys) overlapped with the COVID-19 pandemic, there were no severe impacts from COVID-19 because the entire survey period only had a relatively minor outbreak in Taiwan [44–47].

### Measures

#### Body mass index (BMI) and demographic characteristics

Participants’ BMI was calculated using their self-reported weight (in kg) and height (in cm). BMI was calculated for baseline and follow-up separately. Then, change in BMI was calculated using the follow-up BMI value minus baseline BMI value. Moreover, the participants answered their demographics using multiple choice questions for their degree level (undergraduate or postgraduate), major (see Table 1 for details), gender (male or female), age (in year), monthly income (see Table 1 for more information), current cigarette user (yes or no), current alcohol user (yes or no), and diagnosis of any chronic diseases (yes or no).

**Table 1:** Participants’ characteristics (N = 974)

	n (%)
Age (year)^a^	23.70 (4.31)
Gender (male)	396 (40.7)
Major	
Literal arts	130 (13.3)
Science	82 (8.4)
Management	262 (26.9)
Engineering	119 (12.2)
Electrical engineering and computer science	100 (10.3)
Social science	78 (8.0)
Planning and design	31 (3.2)
Bioscience and biotechnology	35 (3.6)
Medicine	84 (8.6)
Other	53 (5.4)
Degree level (undergraduate)	686 (70.4)
Monthly income	
Below 5000 New Taiwan Dollar (NTD)	145 (14.9)
5000–9999 NTD	105 (10.8)
10,000–14,999 NTD	132 (13.6)
15,000 or above NTD	336 (37.6)
Not reported	226 (23.2)
Current cigarette user (No)	
No	705 (72.4)
Yes	43 (4.4)
Not reported	226 (23.2)
Current alcohol user (No)	
No	662 (68.0)
Yes	86 (8.8)
Not reported	226 (23.2)
Diagnosis of chronic disease (No)	
No	562 (57.7)
Yes	36 (3.7)
Not reported	376 (38.6)

^a^ Reported in Mean (SD).

Note. 1USD ≈ 30 NTD

#### Internalized weight stigma

The Weight Self-Stigma Questionnaire (WSSQ) was used to assess participants’ weight-related self-stigma, referring to internalized weight stigma in this study. The WSSQ contains 12 items with a five-point Likert scale response (1 = completely disagree; 5 = completely agree), and a higher score indicates a higher level of internalized weight stigma. A sample item is “I’ll always go back to being overweight”. The original WSSQ has good psychometric properties [48], and the WSSQ has been translated into different language versions with satisfactory psychometric properties [49–52] including the Chinese version [53]. The present study’s data indicated that the WSSQ used in the present sample had excellent internal consistency (α = 0.93 at baseline; 0.95 at follow-up).

#### Perceived weight stigma

The Perceived Weight Stigma Scale (PWSS) was used to assess participants’ perceived weight stigma. The PWSS contains 10 items with a dichotomous scale response (0 = yes; 1 = no), and a higher score indicates a higher level of perceived weight stigma [54]. A sample item is “Because of your weight, people act as if you are inferior”. The Chinese version of PWSS has good psychometric properties [33, 35, 36, 53]. The present study’s data indicated that the PWSS used in the present sample had excellent internal consistency (Kuder–Richardson Formula 20 [KR-20] = 0.87 at baseline; 0.93 at follow-up).

#### Psychological distress

The Depression, Anxiety, Stress Scale − 21 (DASS-21) was used to assess participants’ psychological distress in three forms: depression, anxiety, and stress. The DASS-21 contains 21 items with a four-point Likert scale response (0 = did not apply to me at all; 3 = applied to me very much), and a higher score indicates a higher level of psychological distress. A sample item is “I found it hard to wind down”. The original DASS-21 has good psychometric properties [55], and the DASS-21 has been translated into different language versions with satisfactory psychometric properties, including the Chinese version [56–58]. The present study’s data indicated that the DASS-21 used in the present sample had excellent internal consistency (α = 0.96 at baseline; 0.98 at follow-up).

### Data analysis

Before the main analyses were performed, missing values were evaluated for whether they were missing completely at random (MCAR) via Little’s MCAR test [59]. If MCAR is not supported, the missing values were evaluated if they were missing at random (MAR) via independent t-tests on the studied variables at baseline. More specifically, if significant differences in the studied variables at baseline were identified between the participants who were retained in the study or lost to follow-up, the MAR is not supported. Findings of the main analyses are considered not having serious biases when the loss to follow-up is less than 60% under either MCAR or MAR condition [60]. Additionally, multiple imputation was used to impute the missing values, given that it provides more robust results than the dataset without imputation when MCAR or MAR is supported [61].

Most demographics of the participants were analyzed using frequency and percentage with age using mean and SD. Afterward, changes in BMI, internalized weight stigma, perceived weight stigma, and psychological distress were calculated using the follow-up scores (or values) minus the baseline scores (or values). Therefore, a positive change score indicated greater scores in follow-up, whereas a negative change score indicated smaller scores in follow-up. The change scores were then used to examine the magnitude of their associations via Pearson correlation coefficients.

Hierarchical regressions were then used to examine how the change in BMI scores could be explained by the changes in weight stigma and psychological distress. More specifically, Model 1 in the hierarchical regression involved two demographic variables (i.e., age and gender) as independent variables with the change in BMI as the dependent variable; Model 2 was based on Model 1 to add changes in perceived weight stigma, internalized weight stigma, and psychological distress as independent variables. Finally, a mediation model was conducted using Hayes’ Process Macro (Model 4) with 5000 bootstrapping samples. In the mediation model, change in perceived weight stigma was treated as the independent variable; change in internalized weight stigma as the mediator; age, gender, and change in psychological distress as covariates; change in BMI as a dependent variable. A significant mediated effect of internalized weight stigma would be supported when the 95% confidence interval in the 5000 bootstrapping samples does not cover 0 [62]. Moreover, given that males and females are likely to have different experiences of weight stigma and weight status, both hierarchical regressions and mediation model were conducted separately for males and females as further subgroup analyses.

The hierarchical regression and mediation models were constructed using change scores instead of using baseline and follow-up scores together because of the following reasons. First, using both baseline and follow-up scores to construct the model may cause the issue of collinearity (e.g., baseline perceived weight stigma and follow-up perceived weight stigma are two highly correlated independent variables in the regression model). Second, using change score could minimize the number of independent variables in the model, and a simpler model is better to satisfy the principal of parsimony in statistical analysis. However, change scores are likely to be attributable to the standard error of measurement. Therefore, significant tests were carried out to ensure the changes are true (i.e., baseline and follow-up scores are significantly different) before constructing the models. In this regard, baseline BMI, percentage overweight (i.e., % of participants with BMI > 24 kg/m^2^), perceived weight stigma, internalized weight stigma, and psychological distress were compared with follow-up BMI, percentage of overweight, perceived weight stigma, internalized weight stigma, and psychological distress using McNamer test (for percentage of overweight) or paired t-tests (for other variables).

## Results

Among the 974 participants (396 males [40.7%]; mean [SD] age = 23.70 [4.31] years), nearly three-fourths were undergraduates (n = 686 [70.4%]). In addition, the top three majors of the present sample were management (n = 262 [26.9%]), liberal arts (n = 130 [13.3%]), and engineering (n = 119 [12.2%]). Most of the participants were not current cigarette users (n = 705 [72.4%]) or current alcohol users (n = 662 [68.0%]). Very few participants self-reported that they had a chronic disease (n = 36 [3.7%]) (Table 1).

Little’s MCAR test was significant (χ^2^ = 103.79; df = 8; p-value < 0.001); therefore, the missing values were not MCAR. The following independent t-tests on the missing values supported that the missing values were MAR (p-values = 0.32 to 0.99). Moreover, the loss to follow-up was 38.6% (376 of 974). Therefore, multiple imputations were used for the following analyses.

Significant differences were found between baseline and follow-up measures in BMI (22.20 vs. 22.54; p-value < 0.001), overweight% (27.9% vs. 30.1%; p-value = 0.003), perceived weight stigma (1.27 vs. 1.71; p-value = 0.002), internalized weight stigma (30.88 vs. 29.46; p-value = 0.001), and psychological distress (13.12 vs. 15.25; p-value = 0.001) (Table 2). The significant differences found between baseline and follow-up scores indicate that the change scores might not be severely influenced by the standard error of measurement.

**Table 2:** Descriptive statistics of studied variables in baseline and follow-up

	Baseline	Follow-up	p-value
Body mass index; M (SD)^a^	22.20 (3.73)	22.54 (3.68)	< 0.001
Overweight; n (%)^b^	255 (27.9)	293 (30.1)	0.003
Perceived weight stigma; M (SD)^a^	1.27 (2.28)	1.71 (2.64)	0.002
Internalized weight stigma; M (SD)^a^	30.88 (10.69)	29.46 (10.49)	0.001
Psychological distress change; M (SD)^a^	13.12 (12.81)	15.25 (14.59)	0.001

^a^ Tested using paired-t tests

^b^ Tested using McNamer test

Pearson correlations (Table 3) showed that changes in perceived weight stigma (r = 0.12; p-value < 0.001) and internalized weight stigma (r = 0.27; p-value < 0.001) were significantly and positively associated with the change in BMI; changes in perceived weight stigma, internalized weight stigma, and psychological distress were significantly and positively associated with each other (r = 0.26 to 0.36; p-values < 0.001).

**Table 3:** Correlations between change scores of studied variables (N = 974)

	Mean	SD	Range			r (p-value)			
				1. Age	2. Gender	3. BMI change	4. Perceived weight stigma change^a^	5. Internalized weight stigma change^b^	6. Psychological distress change^c^
1.	23.70	4.31	20–45	--					
2.	--	--	--	-0.04 (0.28)	--				
3.	0.34	2.29	-10.41, 9.59	-0.02 (0.48)	-0.02 (0.57)	--			
4.	0.50	2.67	-10.00, 10.00	-0.04 (0.25)	0.07 (0.046)	0.12 (< 0.001)	--		
5.	-1.70	8.12	-46.00, 29.00	-0.12 (< 0.001)	-0.02 (0.51)	0.27 (< 0.001)	0.26 (< 0.001)	--	
6.	1.91	12.58	-54.00, 47.00	-0.03 (0.36)	0.04 (0.26)	-0.01 (0.76)	0.32 (< 0.001)	0.36 (< 0.001)	--

BMI = body mass index;

^a^ Assessed using Perceived Weight Stigma Scale

^b^ Assessed using Weight Self-Stigma Questionnaire

^c^ Assessed using Depression, Anxiety, Stress Scale-21

Hierarchical regression models showed that changes in perceived weight stigma (standardized coefficient [β] = 0.09; p-value = 0.01) and internalized weight stigma (β = 0.30; p-value < 0.001) were associated with the change in BMI in a positive direction; change in psychological distress was associated in a negative direction (β = -0.15; p-value < 0.001) (Table 4). In the subgroup analysis of the female subgroup, changes in perceived weight stigma (β = 0.10; p-value = 0.01) and internalized weight stigma (β = 0.32; p-value < 0.001) were associated with the change in BMI in a positive direction; change in psychological distress was associated in a negative direction (β = -0.11; p-value < 0.001). However, in the male subgroup, changes in internalized weight stigma (β = 0.26; p-value < 0.001) and psychological distress (β = -0.20; p-value = 0.001) were associated with change in BMI, while perceived weight stigma was not associated with change in BMI (β = 0.05; p-value < 0.21) (Table 5).

**Table 4:** Changes in body mass index explained by changes in weight stigma and psychological distress

	Coeff. (SE)/ Stand. Coeff. (p-value)	
	Model 1	Model 2
Age	-0.01 (0.02)/ -0.02 (0.47)	0.01 (0.02)/ 0.01 (0.73)
Gender (Ref: female)	-0.09 (0.16)/ -0.02 (0.56)	-0.06 (0.15)/ -0.01 (0.70)
Change in perceived weight stigma	--	0.08 (0.03)/ 0.09 (0.01)
Change in internalized weight stigma	--	0.09 (0.01)/ 0.30 (< 0.001)
Change in psychological distress	--	-0.03 (0.01)/ -0.15 (< 0.001)
F-value (p-value)	0.43 (0.65)	18.54 (< 0.001)
R^2^ (adjusted R^2^)	0.001 (-0.001)	0.09 (0.09)

Coeff.=coefficient; SE = standard error; Stand. Coeff.=standardized coefficient

**Table 5:** Changes in body mass index explained by changes in weight stigma and psychological distress stratified by gender

	Coefficient (SE)/ Standardized coefficient (p-value)			
	Female		Male	
	Model 1	Model 2	Model 1	Model 2
Age	-0.02 (0.02)/-0.04 (0.41)	0.01 (0.02)/0.02 (0.64)	-0.001 (0.03)/-0.002 (0.97)	0.004 (0.03)/0.01 (0.90)
Change in perceived weight stigma		0.09 (0.04)/0.10 (0.02)		0.06 (0.05)/0.07 (0.21)
Change in internalized weight stigma		0.09 (0.01)/0.32 (< 0.001)		0.07 (0.02)/0.26 (< 0.001)
Change in psychological distress		-0.02 (0.01)/-0.11 (0.02)		-0.03 (0.01)/-0.20 (0.001)
F-value (p-value)	0.69 (0.41)	16.84 (< 0.001)	0.002 (0.97)	7.13 (< 0.001)
R^2^ (adjusted R^2^)	0.001 (-0.001)	0.11 (0.10)	0.000 (-0.003)	0.07 (0.06)

**Fig. 1:**
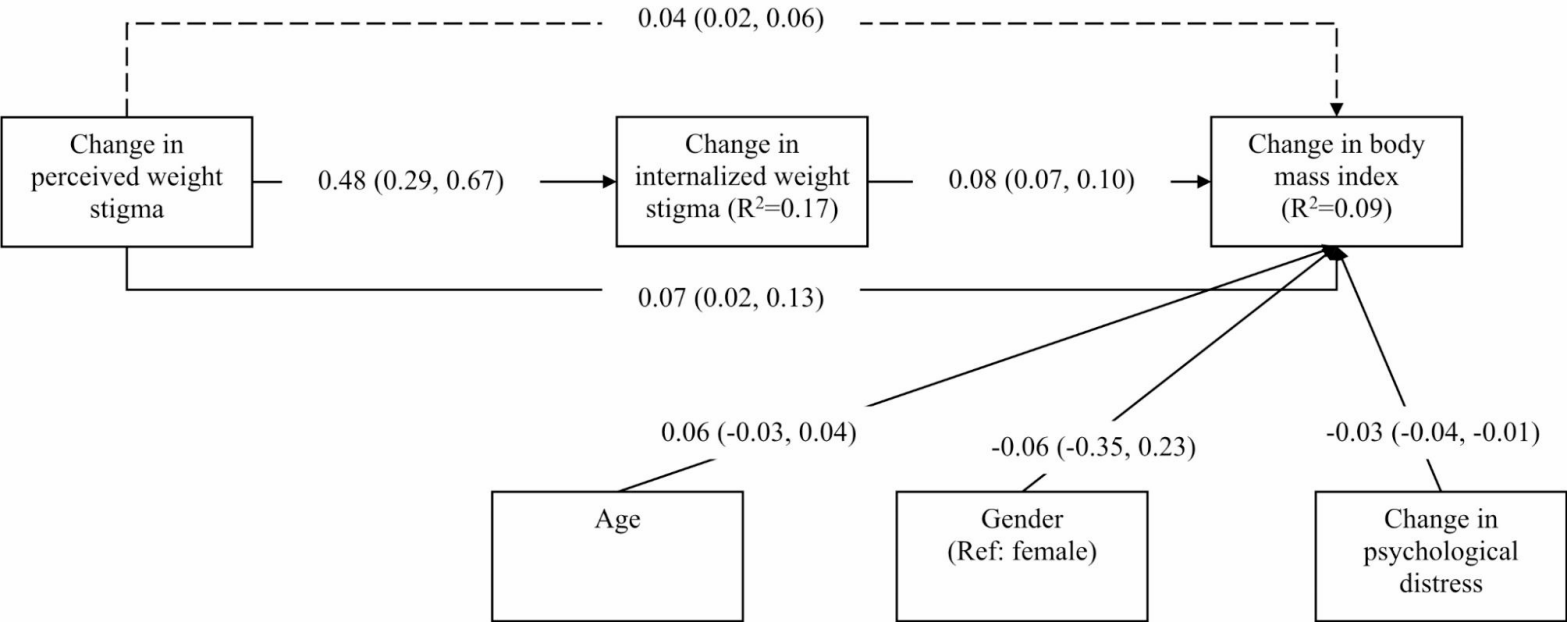
Mediated effects of internalized weight stigma in the association between perceived weight stigma and body mass index. Dashed line indicates indirect effects; solid lines indicate direct effect. Total effect of change in perceived weight stigma to change in body mass index was 0.11. Coefficients reported using unstandardized coefficients with 95% confidence interval in parentheses. Hayes’ Process Model 4 was used with 5000 bootstrapping samples

**Fig. 2:**
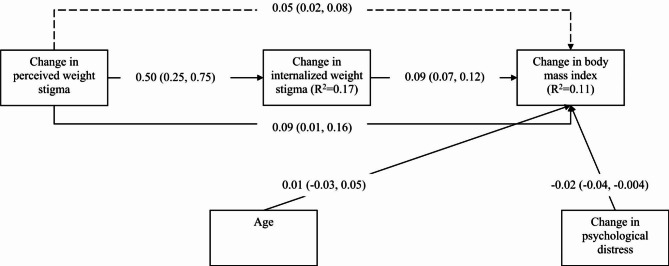
Mediated effects of internalized weight stigma in the association between perceived weight stigma and body mass index among female participants. Dashed line indicates indirect effects; solid lines indicate direct effect. Total effect of change in perceived weight stigma to change in body mass index was 0.13. Coefficients reported using unstandardized coefficients with 95% confidence interval in parentheses. Hayes’ Process Model 4 was used with 5000 bootstrapping samples

**Fig. 3:**
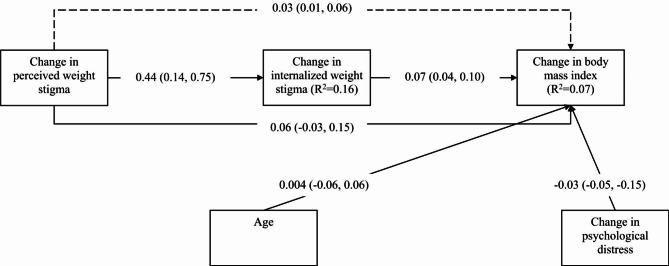
Mediated effects of internalized weight stigma in the association between perceived weight stigma and body mass index among male participants. Dashed line indicates indirect effects; solid lines indicate direct effect. Total effect of change in perceived weight stigma to change in body mass index was 0.09. Coefficients reported using unstandardized coefficients with 95% confidence interval in parentheses. Hayes’ Process Model 4 was used with 5000 bootstrapping samples

The mediation model additionally showed that change in internalized weight stigma was a significant mediator (unstandardized coefficient = 0.04; 95% bootstrapping CI = 0.02, 0.06) in the association between change in perceived weight stigma and change in BMI after controlling age, gender, and change in psychological distress (Fig. 1). Moreover, change in perceived weight stigma had a significantly direct effect on the change in BMI (unstandardized coefficient = 0.07; 95% bootstrapping CI = 0.02, 0.13). Subgroup analysis in the mediation model showed that change in internalized weight stigma remained a significant mediator in both female and male subgroups (Figs. 2 and 3). However, a slight difference was found in the subgroup analysis: change in perceived weight stigma had a significant effect on change in BMI for female participants (unstandardized coefficient = 0.09; 95% bootstrapping CI = 0.01, 0.16) (Fig. 2) but not for male participants (unstandardized coefficient = 0.06; 95% bootstrapping CI = -0.03, 0.75) (Fig. 3).

## Discussion

College students are a high-risk group for physical inactivity and obesity, especially during the process of experiencing independence, developing behaviors, and forming an independent lifestyle right after leaving the prior structured and disciplined environment of secondary schools [3, 63]. Consequently, weight stigma may emerge to play a crucial role in causing adverse biopsychosocial health outcomes, which might in turn, contribute to the correlates of obesity [10]. However, the mechanisms underlying the impact of weight stigma on changes in BMI have been quite underexplored. More specifically, research is needed on the trajectory of weight stigma, from perceived stigma to internalized stigma, and subsequently to stigma’s impact on BMI change. The present study aimed to expand this weight stigma theoretical model by examining the directional association of weight stigma and changes in BMI within a longitudinal study. This study demonstrated that the associations between perceived weight stigma, internalized weight stigma, and changes in BMI were in line with prior findings. Specifically, the results indicated a positive association and temporal relationship between perceived weight stigma and internalized weight stigma, which were consistent with previous research [8, 32, 35]. A positive gradient was identified between perceived weight stigma and internalized weight stigma [38]. As perceived weight stigma contributes to a higher intensity of internalized weight stigma, this dynamic may, in turn, lead to poorer health behavior and health outcomes [43].

Prior studies have identified that weight-related stigma has been found to have negative consequences for health outcomes [13], including aspects of psychological well-being and physical health [36, 38, 64] and biopsychosocial health [16]. Also, weight stigma may cause detrimental harm to health behaviors and health outcomes that could increase the risk of weight gain [65]. Individuals with high perceived weight stigma were prone to engage in obesity-promoting behaviors [66], such as problematic eating [67], avoidance of dieting [17, 68], and avoidance of physical activity [69]. Internalized weight stigma is also a significant predictor of weight-related health behaviors, including reduced participation in physical activity [70], poorer weight loss maintenance [71], and noncompliance with nutritional recommendations [72]. As a result, internalized weight stigma ultimately predicts poorer weight loss maintenance. However, there is very little evidence of the association between weight stigma and actual changes in BMI. In an effort to fill this research gap, the present study demonstrated that both perceived and internalized weight stigma positively related to the prospective change in BMI. Specifically, through the subgroup analysis, there gender difference was shown on the impact of weight stigma on the changes of BMI. The changes in internalized weight stigma remained a significant mediator in both female and male subgroups; however, change in perceived weight stigma had a significant effect on change in BMI for female participants only. The gender difference may be explained by the Objectification Theory [73, 74]. That is, society teaches self-objectification to women, such that how they are perceived as viewed by society becomes internalized, which makes perceived and internalized weight stigma more closely linked, especially for women during the mate-choice period.

Greater weight stigma corresponded with greater change in BMI. The result is consistent with prior studies indicating that problematic eating [67], avoidance of dieting [17, 68], and avoidance of physical activity [69] could be the consequential behavior of weight stigma that causes a change in BMI. In addition, the present study found that perceived weight stigma and internalized weight stigma were both positively related to changes in psychological distress, which indicated that weight stigma had a crucial impact on the quality of psychological health. Such findings were consistent with prior literature that identified higher perceived weight stigma [75] and internalized weight stigma [17] as both associated with poorer mental health outcomes, with internalized weight stigma having a greater impact on personal psychological health than perceived weight stigma [76]. More specifically, internalized weight stigma was significantly and positively associated with depressive symptoms [31, 77]. Also, perceived weight stigma accounted for 27% of the expected association between greater physiological dysregulation and overweight/obesity [78]. Other studies specified the positive associations between weight stigma and psychological distress, including depression and anxiety [16, 32, 79]; moreover, one study depicted a significant indirect effect of internalized weight stigma in the relationship between perceived weight stigma and depression and anxiety [80], potentially increasing body weight [81].

Psychological distress could provoke weight gain through maladaptive coping strategies, up-regulation of perceived appetite, and emotional eating and binge eating, leading to a vicious circle of further weight gain and continued subsequent distress [82]. The present study supported the mechanism whereby weight stigma may lead to changes in BMI, a possibly obesogenic process, suggesting an ultimately vicious cycle of weight stigma, psychological distress, changes in BMI, and risk of long-term obesity.

Despite several strengths of the current study, including sufficient sample size, a longitudinal approach to trace changes over time, and controlling confounding factors that may affect mediation analysis, there are some limitations in the present study. First, the studied sample was assessed through a web survey, limiting generalizability. Second, responses to the self-report web survey containing questions about weight stigma and BMI could be influenced by social desirability or social approval. Moreover, the follow-up study only lasted for three months; longitudinal effects should be examined over longer time frames in future studies.

In sum, after carefully reviewing the literature for a better understanding of the impact of weight stigma on various health outcomes across physical, psychological, biopsychosocial, and behavioral domains, the present study investigated a theoretical trajectory illustrating how weight stigma is perceived, internalized, and contributes to changes in BMI, the essential cause of obesity, within the context of Asian culture. The findings in this study may help victims of weight stigma better recognize these perceiving-internalizing processes of weight-related perceptions and the consequences on their health. As more data are assembled to replicate these associations, more consistent findings across diverse samples may be of help in devising means of mitigating the harm of weight stigma on health. While Western countries (like the US, Canada, the UK, and ones in Europe) have awarded and called for initiatives to address the issue of weight stigma [38], the same actions should be advocated and recognized in other countries as well. A clearer understanding of weight stigma and its consequences is essential to solving challenges in the field of weight-related health.

## Data Availability

The datasets generated during and/or analyzed during the current study are available from the corresponding author on reasonable request.

## References

[1] Afshin A, Forouzanfar MH (2017). Health effects of overweight and obesity in 195 countries over 25 years. N Engl J Med.

[2] Reilly JJ, Hughes AR, Gillespie J, Malden S, Martin A (2019). Physical activity interventions in early life aimed at reducing later risk of obesity and related non-communicable Diseases: a rapid review of systematic reviews. Obes Rev.

[3] Ráthonyi G, Takács V, Szilágyi R, Bácsné Bába É, Müller A, Bács Z, Harangi-Rákos M, Balogh L, Ráthonyi-Odor K (2021). Your physical activity is in your hand-objective activity tracking among University students in Hungary, one of the most obese countries in Europe. Front Public Health.

[4] Wang C, Lizardo O, Hachen DS (2021). Using fitbit data to examine factors that affect daily activity levels of college students. PLoS ONE.

[5] Clemente FM, Nikolaidis PT, Martins FM, Mendes RS (2016). Physical activity patterns in university students: do they follow the public health guidelines?. PLoS ONE.

[6] Yeh CJ, Chang HY, Pan WH (2011). Time trend of obesity, the metabolic syndrome and related dietary pattern in Taiwan: from NAHSIT 1993–1996 to NAHSIT 2005–2008. Asia Pac J Clin Nutr.

[7] Wong TJ, Yu T (2022). Trends in the distribution of body mass index, waist circumference and prevalence of obesity among Taiwanese adults, 1993–2016. PLoS ONE.

[8] Bidstrup H, Brennan L, Kaufmann L, de la Piedad Garcia X (2022). Internalised weight stigma as a mediator of the relationship between experienced/perceived weight stigma and biopsychosocial outcomes: a systematic review. Int J Obes (Lond).

[9] Puhl RM, Heuer CA (2009). The stigma of obesity: a review and update. Obes (Silver Spring).

[10] Papadopoulos S, Brennan L (2015). Correlates of weight stigma in adults with overweight and obesity: a systematic literature review. Obes (Silver Spring).

[11] Charlesworth TES, Banaji MR (2019). Patterns of implicit and explicit attitudes: I. long-term change and stability from 2007 to 2016. Psychol Sci.

[12] Brewis A, SturtzSreetharan C, Wutich A (2018). Obesity stigma as a globalizing health challenge. Global Health.

[13] Pearl RL, Puhl RM (2018). Weight bias internalization and health: a systematic review. Obes Rev.

[14] Tomiyama AJ, Carr D, Granberg EM, Major B, Robinson E, Sutin AR, Brewis A (2018). How and why weight stigma drives the obesity ‘epidemic’ and harms health. BMC Med.

[15] Puhl RM, Brownell KD (2003). Psychosocial origins of obesity stigma: toward changing a powerful and pervasive bias. Obes Rev.

[16] Puhl R, Suh Y (2015). Health consequences of weight stigma: implications for obesity prevention and treatment. Curr Obes Rep.

[17] Emmer C, Bosnjak M, Mata J (2020). The association between weight stigma and mental health: a meta-analysis. Obes Rev.

[18] Throop EM, Skinner AC, Perrin AJ, Steiner MJ, Odulana A, Perrin EM (2014). Pass the popcorn: obesogenic behaviors and stigma in children’s movies. Obes (Silver Spring).

[19] Stanford FC, Tauqeer Z, Kyle TK (2018). Media and its influence on obesity. Curr Obes Rep.

[20] Tomiyama AJ (2019). Stress and obesity. Annu Rev Psychol.

[21] Decker KM, Thurston IB, Kamody RC (2018). The mediating role of internalized weight stigma on weight perception and depression among emerging adults: exploring moderation by weight and race. Body Image.

[22] Durso LE, Latner JD (2008). Understanding self-directed stigma: development of the weight bias internalization scale. Obes (Silver Spring).

[23] Zuba A, Warschburger P (2017). The role of weight teasing and weight bias internalization in psychological functioning: a prospective study among school-aged children. Eur Child Adolesc Psychiatry.

[24] Trojanowski PJ, Breithaupt L, Negi S, Wonderlich J, Fischer S (2020). Lack of guilt, shame, and remorse following weight stigma expression: a real-time assessment pilot study. PeerJ.

[25] Callahan D (2013). Children, stigma, and obesity. JAMA Pediatr.

[26] Puhl RM, Moss-Racusin CA, Schwartz MB, Brownell KD (2008). Weight stigmatization and bias reduction: perspectives of overweight and obese adults. Health Educ Res.

[27] Grogan S (2021). Body image: understanding body dissatisfaction in men, women, and children.

[28] Weinberger NA, Kersting A, Riedel-Heller SG, Luck-Sikorski C (2016). Body dissatisfaction in individuals with obesity compared to normal-weight individuals: a systematic review and meta-analysis. Obes Facts.

[29] Ashmore JA, Friedman KE, Reichmann SK, Musante GJ (2008). Weight-based stigmatization, psychological distress, & binge eating behavior among obese treatment-seeking adults. Eat Behav.

[30] Hunger JM, Tomiyama AJ (2014). Weight labeling and obesity: a longitudinal study of girls aged 10 to 19 years. JAMA Pediatr.

[31] O’Brien KS, Latner JD, Puhl RM, Vartanian LR, Giles C, Griva K, Carter A (2016). The relationship between weight stigma and eating behavior is explained by weight bias internalization and psychological distress. Appetite.

[32] Wu YK, Berry DC (2018). Impact of weight stigma on physiological and psychological health outcomes for overweight and obese adults: a systematic review. J Adv Nurs.

[33] Xu P, Chen JS, Chang YL, Wang X, Jiang X, Griffiths MD, Pakpour AH, Lin CY (2022). Gender differences in the associations between physical activity, smartphone use, and weight stigma. Front Public Health.

[34] Kamolthip R, Fung XCC, Lin CY, Latner JD, O’Brien KS (2021). Relationships among physical activity, health-related quality of life, and weight stigma in children in Hong Kong. Am J Health Behav.

[35] Lin CY, Strong C, Latner JD, Lin YC, Tsai MC, Cheung P (2020). Mediated effects of eating disturbances in the association of perceived weight stigma and emotional distress. Eat Weight Disord.

[36] Cheng MY, Wang SM, Lam YY, Luk HT, Man YC, Lin CY (2018). The relationships between weight bias, perceived weight stigma, eating behavior, and psychological distress among undergraduate students in Hong Kong. J Nerv Ment Dis.

[37] Tylka TL, Annunziato RA, Burgard D, Daníelsdóttir S, Shuman E, Davis C, Calogero RM. The weight-inclusive versus weight-normative approach to health: evaluating the evidence for prioritizing well-being over weight loss. J Obes. 2014;983495. 10.1155/2014/983495. 10.1155/2014/983495 PMC4132299 25147734

[38] Forbes Y, Donovan C (2019). The role of internalised weight stigma and self-compassion in the psychological well‐being of overweight and obese women. Aust Psychol.

[39] Pearl RL, Puhl RM, Dovidio JF (2015). Differential effects of weight bias experiences and internalization on exercise among women with overweight and obesity. J Health Psychol.

[40] Gee GC, Ro A, Gavin A, Takeuchi DT (2008). Disentangling the effects of racial and weight discrimination on body mass index and obesity among Asian americans. Am J Public Health.

[41] Tomiyama AJ (2014). Weight stigma is stressful. A review of evidence for the cyclic obesity/weight-based stigma model. Appetite.

[42] Pearl RL, Puhl RM, Lessard LM, Himmelstein MS, Foster GD (2021). Prevalence and correlates of weight bias internalization in weight management: a multinational study. SSM Popul Health.

[43] Puhl RM, Lessard LM, Himmelstein MS, Foster GD (2021). The roles of experienced and internalized weight stigma in healthcare experiences: perspectives of adults engaged in weight management across six countries. PLoS ONE.

[44] Chung GK, Strong C, Chan YH, Chung RY, Chen JS, Lin YH, Huang RY, Lin CY, Ko NY (2022). Psychological distress and protective behaviors during the covid-19 pandemic among different populations: Hong Kong general population, Taiwan healthcare workers, and Taiwan outpatients. Front Med (Lausanne).

[45] Huang WY, Tsang HWH, Wang SM, Huang YC, Chen YC, Cheng CH, Chen CY, Chen JS, Chang YL, Huang RY, Lin CY, Potenza MN, Pakpour AH (2022). Effectiveness of using calligraphic activity to treat people with schizophrenia: a randomized controlled trial in Southern Taiwan. Ther Adv Chronic Dis.

[46] Kuo YJ, Chen YP, Wang HW, Liu CH, Strong C, Saffari M, Ko NY, Lin CY, Griffiths MD (2021). Community outbreak moderates the association between COVID-19-related behaviors and COVID-19 fear among older people: a one-year longitudinal study in Taiwan. Front Med (Lausanne).

[47] Lin CY, Fan CW, Ahorsu DK, Lin YC, Weng HC, Griffiths MD (2022). Associations between vaccination and quality of life among Taiwan general population: a comparison between COVID-19 vaccines and Flu vaccines. Hum Vaccin Immunother.

[48] Lillis J, Luoma JB, Levin ME, Hayes SC (2010). Measuring weight self-stigma: the weight self-stigma questionnaire. Obes (Silver Spring).

[49] Gan WY, Tung SEH, Kamolthip R, Ghavifekr S, Chirawat P, Nurmala I, Chang YL, Latner JD, Huang RY, Lin CY (2022). Evaluation of two weight stigma scales in Malaysian university students: weight self-stigma questionnaire and perceived weight stigma scale. Eat Weight Disord.

[50] Lin CY, Imani V, Cheung P, Pakpour AH (2020). Psychometric testing on two weight stigma instruments in Iran: Weight Self-Stigma Questionnaire and Weight Bias internalized Scale. Eat Weight Disord.

[51] Lin KP, Lee ML. Validating a Chinese version of the weight self-stigma Questionnaire for use with obese adults. Int J Nurs Pract. 2017;23(4). 10.1111/ijn.12537. 10.1111/ijn.12537 28303628

[52] Maïano C, Aimé A, Lepage G, ASPQ Team, Morin AJS (2019). Psychometric properties of the Weight Self-Stigma Questionnaire (WSSQ) among a sample of overweight/obese french-speaking adolescents. Eat Weight Disord.

[53] Pakpour AH, Tsai MC, Lin YC, Strong C, Latner JD, Fung XCC, Lin CY, Tsang HWH (2019). Psychometric properties and measurement invariance of the Weight Self-Stigma Questionnaire and Weight Bias internalization scale in children and adolescents. Int J Clin Health Psychol.

[54] Nadhiroh SR, Nurmala I, Pramukti I, Tivany ST, Tyas LW, Zari AP (2022). Weight stigma in Indonesian young adults: validating the Indonesian versions of the weight self-stigma questionnaire and perceived weight stigma scale. Asian J Soc Health Behav.

[55] Lovibond SH, Lovibond PF (1995). Manual for the Depression anxiety stress scales.

[56] Cao C-h, Laio X-l, Gamble JH, Li L-l, Jian X-Y, Li X-D, Griffiths MD, Chen I-H, Lin C-Y (2023). Evaluating the Psychometric properties of the Chinese depression anxiety stress scale for Youth (DASS-Y) and DASS-21. Child Adolesc Psychiatry Ment Health.

[57] Chen IH, Chen CY, Liao XL (2023). Psychometric properties of the Depression, anxiety, and stress scale (DASS-21) among different Chinese populations: a cross-sectional and longitudinal analysis. Acta Psychol (Amst).

[58] Cao CH, Liao XL, Jiang XY, Li XD, Chen IH, Lin CY (2023). Psychometric evaluation of the depression, anxiety, and stress scale-21 (DASS-21) among Chinese primary and middle school teachers. BMC Psychol.

[59] Little RJ (1988). A test of missing completely at random for multivariate data with missing values. J Am Stat Assoc.

[60] Kristman V, Manno M, Côté P (2004). Loss to follow-up in cohort studies: how much is too much?. Eur J Epidemiol.

[61] Sterne JA, White IR, Carlin JB, Spratt M, Royston P, Kenward MG, Wood AM, Carpenter JR (2009). Multiple imputation for missing data in epidemiological and clinical research: potential and pitfalls. BMJ.

[62] Hu YL, Chang CC, Lee CH, Liu CH, Chen YJ, Su JA, Lin CY, Griffiths MD (2023). Associations between Affiliate Stigma and Quality of Life among caregivers of individuals with Dementia: mediated roles of Caregiving Burden and Psychological Distress. Asian J Soc Health Behav.

[63] Gallardo-Escudero A, Muñoz Alférez MJ, Planells del Pozo EM, López Aliaga I (2014). La Etapa Universitaria no favorece El estilo de vida saludable en las estudiantes granadinas [The university stage does not favor the healthy life style in women students from Granada]. Nutr Hosp.

[64] Hunger JM, Major B (2015). Weight stigma mediates the association between BMI and self-reported health. Health Psychol.

[65] Major B, Hunger JM, Bunyan DP, Miller CT (2014). The ironic effects of weight stigma. J Exp Soc Psychol.

[66] Jackson SE, Beeken RJ, Wardle J (2014). Perceived weight discrimination and changes in weight, waist circumference, and weight status. Obes (Silver Spring).

[67] Durso LE, Latner JD, Hayashi K (2012). Perceived discrimination is associated with binge eating in a community sample of non-overweight, overweight, and obese adults. Obes Facts.

[68] Puhl RM, Brownell KD (2006). Confronting and coping with weight stigma: an investigation of overweight and obese adults. Obes (Silver Spring).

[69] Han S, Agostini G, Brewis AA, Wutich A (2018). Avoiding exercise mediates the effects of internalized and experienced weight stigma on physical activity in the years following bariatric Surgery. BMC Obes.

[70] Mensinger J, Meadows A (2017). Internalized weight stigma mediates and moderates physical activity outcomes during a healthy living program for women with high body mass index. Psychol Sport Exerc.

[71] Lillis J, Thomas JG, Levin ME, Wing RR (2020). Self-stigma and weight loss: the impact of fear of being stigmatized. J Health Psychol.

[72] Raves DM, Brewis A, Trainer S, Han SY, Wutich A (2016). Bariatric Surgery patients’ perceptions of weight-related stigma in healthcare settings impair post-surgery dietary adherence. Front Psychol.

[73] Fredrickson BL, Roberts TA (1997). Objectification theory: toward understanding women’s lived experiences and mental health risks. Psychol Women Q.

[74] Daniels EA, Zurbriggen EL, Monique Ward L (2020). Becoming an object: a review of self-objectification in girls. Body Image.

[75] Lee KM, Hunger JM, Tomiyama AJ (2021). Weight stigma and health behaviors: evidence from the eating in America Study. Int J Obes (Lond).

[76] Hatzenbuehler ML, Keyes KM, Hasin DS (2009). Associations between perceived weight discrimination and the prevalence of psychiatric disorders in the general population. Obes (Silver Spring).

[77] Zhu X, Smith RA, Buteau E (2022). A meta-analysis of weight stigma and health behaviors. Stigma Health.

[78] Daly M, Sutin AR, Robinson E (2019). Perceived weight discrimination mediates the prospective association between obesity and physiological dysregulation: evidence from a population-based cohort. Psychol Sci.

[79] Puhl RM, Heuer CA (2010). Obesity stigma: important considerations for public health. Am J Public Health.

[80] Magallares A, Bolaños-Rios P, Ruiz-Prieto I, Benito de Valle P, Irles JA, Jáuregui-Lobera I (2017). The mediational effect of weight self-stigma in the relationship between blatant and subtle discrimination and depression and anxiety. Span J Psychol.

[81] Dixon JB (2010). The effect of obesity on health outcomes. Mol Cell Endocrinol.

[82] Hemmingsson E (2014). A new model of the role of psychological and emotional distress in promoting obesity: conceptual review with implications for treatment and prevention. Obes Rev.

